# Influence of L-lactate and low glucose concentrations on the metabolism and the toxin formation of *Clostridioides difficile*

**DOI:** 10.1371/journal.pone.0244988

**Published:** 2021-01-07

**Authors:** Julia Danielle Hofmann, Rebekka Biedendieck, Annika-Marisa Michel, Dietmar Schomburg, Dieter Jahn, Meina Neumann-Schaal

**Affiliations:** 1 Department of Bioinformatics and Biochemistry, Technische Universität Braunschweig, Braunschweig, Germany; 2 Braunschweig Integrated Centre of Systems Biology (BRICS), Braunschweig, Germany; 3 Institute of Microbiology, Technische Universität Braunschweig, Braunschweig, Germany; 4 Leibniz Institute DSMZ—German Collection of Microorganisms and Cell Cultures, Braunschweig, Germany; Dartmouth College, UNITED STATES

## Abstract

The virulence of *Clostridioides difficile* (formerly *Clostridium difficile*) is mainly caused by its two toxins A and B. Their formation is significantly regulated by metabolic processes. Here we investigated the influence of various sugars (glucose, fructose, mannose, trehalose), sugar derivatives (mannitol and xylitol) and L-lactate on toxin synthesis. Fructose, mannose, trehalose, mannitol and xylitol in the growth medium resulted in an up to 2.2-fold increase of secreted toxin. Low glucose concentration of 2 g/L increased the toxin concentration 1.4-fold compared to growth without glucose, while high glucose concentrations in the growth medium (5 and 10 g/L) led to up to 6.6-fold decrease in toxin formation. Transcriptomic and metabolic investigation of the low glucose effect pointed towards an inactive CcpA and Rex regulatory system. L-lactate (500 mg/L) significantly reduced extracellular toxin formation. Transcriptome analyses of the later process revealed the induction of the lactose utilization operon encoding lactate racemase (*larA*), electron confurcating lactate dehydrogenase (*CDIF630erm_01321*) and the corresponding electron transfer flavoprotein (*etfAB*). Metabolome analyses revealed L-lactate consumption and the formation of pyruvate. The involved electron confurcation process might be responsible for the also observed reduction of the NAD^+^/NADH ratio which in turn is apparently linked to reduced toxin release from the cell.

## Introduction

The virulence of the pathogenic, anaerobic bacterium *Clostridioides difficile* (formerly *Clostridium difficile*) is mainly attributed to its two large clostridial toxins TcdA and TcdB. Both toxins have a glycosyltransferase activity and modify GTP-binding proteins of the Ras superfamily via glycozylation. These small regulatory proteins, which are involved in cytoskeleton rearrangement, are irreversible inactivated, which leads to a disaggregation of the microfilaments [1, 2]. The symptoms range from mild diarrhea to life-threatening colitis. The formation of the toxins by *C*. *difficile* is strongly influenced by its nutritional status and the corresponding metabolic networks. The amino acids proline and cysteine are the favored substrates of *C*. *difficile*. Increased availability in the medium leads to reduced toxin production in a dose-dependent manner [3–5]. In complex medium glucose (1–10 g/L) and other rapidly metabolizable sugars also lead to lower toxin production [6, 7]. In an amino acid-based minimal medium, supplementation of glucose (0.02–2 g/L) increases formation of toxins [7]. The background of this discrepancy is not fully understood.

Due to the high antibiotic resistance of this pathogen [8], changing the nutritional status could be a possibility to influence its growth or toxin production. This could be achieved by certain diets for the patients where the nutrient status in the gut is changed. Recent studies showed that high-fat and high-fat/high-protein diets leading to severe *C*. *difficile* infections in mice. In contrast, a high-carbohydrate diet showed a protective role against the infection [9]. In another study, mice treated with microbiota-accessible carbohydrates showed a suppressed burden of *C*. *difficile* infection by increased microbiota diversity and high levels of short chain fatty acids [10]. Another recent example is trehalose which is used as a food additive in human diet. It has been identified as one possible reason for the worldwide spread of hypervirulent *C*. *difficile* strains due to their ability to metabolize low concentrations of this disaccharide [11].

Experiments showed that extracellular toxins were predominantly found in the stationary phase [12, 13], which is linked to the major changes in metabolism during the transition from the logarithmic growth phase. At that point, the metabolism shifts from the degradation of amino acids via Stickland reactions to butanoate fermentation and further to lactate formation. The toxins accumulate extracellularly in the later stationary phase when lactate is secreted.

In this work, we investigated the influence of various sugars, sugar alcohols, and lactate on the overall metabolism and toxin formation in *C*. *difficile*. For this purpose targeted and non-targeted metabolomic approaches were combined with transcriptomic analyses.

## Materials and methods

### Strain and cultivation conditions

Studies were performed with *C*. *difficile* 630Δ*erm* (DSM28645) [14] obtained from the German Collection of Microorganisms and Cell Cultures (DSMZ, Braunschweig, Germany). The gene *erm* encodes a methylase which causes conformational changes of the ribosome so that the binding of erythromycin is reduced [14]. *C*. *difficile* 630Δ*erm*, an erythromycin-sensitive derivative of the wild-type strain 630 [14], is the most used model strain for basic molecular and genetic investigations of *C*. *difficile*. Media used in this study were the defined medium CDMM, which contains glucose and casamino acids as carbon and energy sources [12, 15] and CDMM without glucose (CAM). All other parameters, such as amino acid concentrations, pH, buffering capacity and trace salt supplementation were the same in CAM and CDMM [12, 15]. All pre-cultures were cultivated in CDMM. Main cultures were cultivated in CAM as reference as well as in CDMM and CAM with different additives. For the initial screening experiment, CAM was supplemented with different components of human nutrition (glucose, fructose, mannose, trehalose, mannitol, xylitol, L-lactate) in concentrations between 500 mg/L and 10 g/L. For the following experiments, CDMM as well as CAM were used. Additionally, CAM was supplemented as indicated with 500 mg/L L-lactate (named as CAM+L). Casamino acids were obtained from Merck (Darmstadt, Germany) while the same lot was used throughout all experiments. Growth was monitored in Hungate glass tubes at 600 nm using a WPA CO8000 Cell density meter (Biochrom, UK). Before inoculating the main cultures, cells of the pre-cultures were transferred twice with a dilution of 1:1,000 and grown for approximately 24 h under anaerobic conditions. The pre-cultures for main-culture inoculation were cultivated until the late exponential phase was reached. Main cultures were inoculated each time to a starting OD_600nm_ of ~ 0.01. Growth experiments were performed at 37°C. Samples were taken at the middle of the exponential phase (½ OD_max_) and in the beginning of the stationary phase (OD_max_) as indicated. All experiments were performed in four independent replicates.

### Toxin quantification

Quantification of toxins A and B in the supernatant was performed using the TGC-E002-1 ELISA (tgcBIOMICS GmbH, Bingen, Germany) as described before [12]. For better comparability between the cultures, the amount of toxin in ng/mL obtained from the ELISA was related to the cell dry weight of *C*. *difficile* per mL culture. For the quantification of the intracellular toxin content cells of 25 mL culture were harvested by centrifugation (10 min, 14,000 x g, 4°C) at the different time points, washed once with 2 mL sterile, anaerobic, cold phosphate buffered saline (4°C) and immediately frozen in liquid nitrogen. For the extraction of the intracellular toxins the precipitated cells were resuspended in 800 μL dilution buffer of the ELISA kit and mixed with 600 mg glass beads. Cells were lysed using a Mixer Mill MM 400 (Retsch GmbH, Haan, Germany). The procedure included three cycles of homogenization and complete defrosting between the cycles. In one cycle, the samples were homogenized three times two minutes with 20 seconds break in between at < -20°C. The lysate was centrifuged (5 min, 16,000 x g, 4°C) and the supernatant was stored on ice until toxin quantification by the ELISA kit.

### Cell harvest

Samples for metabolome, NAD^+^/NADH and microarray analyses were harvested anaerobically at the middle of the exponential phase and at the beginning of the stationary phase by centrifugation (10 min, 14,000 x g, 4°C). The precipitated cells for the analysis of intracellular metabolites were immediately quenched in pre-cooled isotonic sodium chloride/methanol (50% (v/v), -32°C) by resuspension. Cells were centrifuged again (5 min, 14,000 x g, -20°C) to remove the quenching solution and were frozen in liquid nitrogen. For the analysis of NAD^+^ and NADH, cells were anaerobically washed once with 5 mL sterile, anaerobic, cold phosphate buffered saline (4°C). After centrifugation (5 min, 14,000 x g, 4°C) the cells were frozen in liquid nitrogen and stored at -80°C for a short time. For the transcriptome analyses, the precipitated cells were immediately frozen in liquid nitrogen. The supernatant for extracellular metabolome analysis was sterile-filtered and frozen at -80°C.

### Extraction of intracellular and extracellular metabolites

To extract the intracellular metabolites, the precipitated cells were re-suspended in 1.5 mL methanol containing 0.4 μg/mL ribitol per 10 mg cell dry weight. The suspension was incubated in an ultrasonic bath for 15 min at 70°C. After cooling on ice, the same volume of water was added and the samples were vigorously mixed for one minute. Subsequently, chloroform was added in a volume of 2/3 of that of methanol and the samples were again vigorously mixed for one minute. The phases were separated by centrifugation (5 min, 14,000 x g, 4°C). One mL of the polar phase was transferred in a glass vial and dried under vacuum. For the analysis of non-volatile extracellular metabolites 10 μL of the culture supernatant was supplemented with 500 μL ethanol containing 4 μg/mL ribitol as internal standard. The samples were dried under vacuum.

### Analysis of intracellular and extracellular metabolites

GC-MS measurements on non-volatile compounds were performed after online derivatization. In a two-step derivatization protocol the dried samples were methoxymated using a methoxyamine hydrochloride solution with a concentration of 20 mg/mL in pyridine. In the second step silylation was achieved by applying N-methyl-N-(trimethylsilyl)-trifluoroacetamide (MSTFA). Obtained samples were measured using a Leco Pegasus 4D GCxGC TOFMS (Leco Instrumente, Mönchengladbach, Germany) combined with an Agilent 7890A GC (Agilent Technologies, Böblingen, Germany) and a Gerstel MPS 2 XL Twister autosampler (Gerstel, Mühlheim a. d. Ruhr, Germany) [12, 16, 17].

Volatile compounds were determined following an ether extraction into 200 μL *tert*-methylbutylether from 400 μL of the culture supernatant which were mixed with 60 μL of a HPLC-grade sulfuric acid solution and 600 μL of an internal standard solution of *o*-cresol for this purpose. After vigorously mixing and centrifugation (5 min, 16,000 x g, 4°C), the ether phase was transferred into a GC-MS vial. The compounds were measured on an Agilent VF-WAXms column (0.25 mm x 30 m, Agilent, Santa Clara, USA) on a Thermo DSQ II gas chromatograph equipped with a liner and quadrupol mass spectrometer as described before [12]. Free amino acids were measured on a 1260 Infinity HPLC system equipped with a fluorescence detector (Agilent Technologies, Waldbronn, Germany) and a Poroshell HPH-C18 separation column (4.6 x 100 mm, particle size 2.7 mm; Agilent Technologies) as described previously [13, 18, 19]. Prior to analysis, ammonium was precipitated from the samples by a 1:1 dilution with sodium tetraphenylborate (250 mM). Quantification of L- and D-lactate in the culture supernatant was performed using the L-lactate and D-lactate enzyme assay (R-Biopharm, Darmstadt, Germany) according to the supplier’s manual after 24 h of growth. Quantification of D-Glucose in the medium, the exponential and the stationary phase was performed using the D-glucose enzyme assay (R-Biopharm, Darmstadt, Germany) according to the supplier’s manual.

### NAD^+^/NADH extraction and quantification

The extraction was done aerobically. Cells were lysed in 600 μL extraction buffer from the NAD^+^/NADH Quantitation Kit (Sigma-Aldrich, Darmstadt, Germany) and 300 μL chloroform supplemented with 400 mg glass beads in a Mixer Mill MM 400 (Retsch GmbH, Haan, Germany). The procedure included three cycles of homogenization with 20 seconds break in between at < -20°C. After thawing, the samples were centrifuged (5 min, 16,000 x g, 4°C). Quantification of NAD^+^/NADH was conducted with the NAD^+^/NADH Quantitation Kit obtained from Sigma (Sigma-Aldrich, Darmstadt, Germany) according to the supplier’s manual.

### RNA extraction, microarray experiment and data analysis

Bacterial RNA extraction, performance of the microarray experiment and the data analysis was conducted as described previously [13]. The complete experimental data sets were deposited in the GEO database with the accession number GSE149911 for the datasets of CDMM, CAM+L and CAM.

### Statistics

The normal distribution of the data was verified by a Shapiro-Wilk test using the OriginPro 2016 software.

Significant changes were analyzed by Wilcoxon-Mann-Whitney tests using the OriginPro 2016 software or the TigrMev software (version 4.6.2). The fold changes of the metabolic data with the adjusted p-values as well as the log2 fold changes of the transcriptomic data with the adjusted p-values can be found in the supplementary files.

## Results

### Influence of sugars and sugar alcohols on growth and toxin secretion by *C*. *difficile*

To analyze the influence of different food additives on growth and toxin formation of *C*. *difficile* 630Δ*erm*, the bacterium was grown in casamino acids medium (CAM) with the addition of various common sugars and sugar alcohols (fructose, glucose, mannose, trehalose, mannitol and xylitol) and the common nutritional sugar-derived metabolite L-lactate as initial screening experiment. The addition of glucose, fructose, mannose or mannitol increased growth in a comparable manner (up to 1.8-fold), while trehalose increased growth to a lower extent (1.3-fold). Addition of xylitol and L-lactate did not significantly influence the growth behavior of *C*. *difficile* under the employed conditions (S1 Fig).

**Fig 1:**
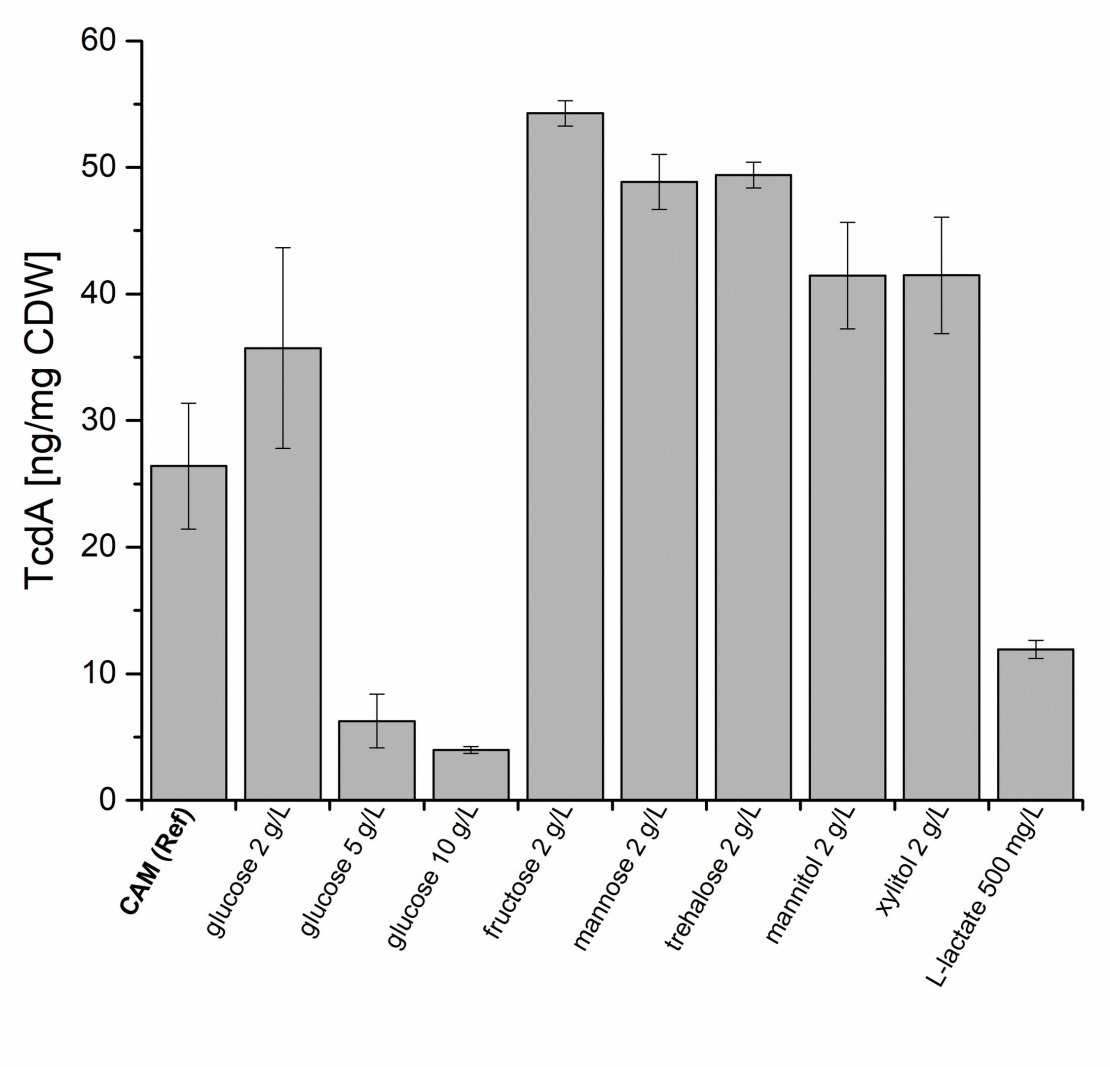
Extracellular concentration of toxin A (TcdA) excreted by *C*. *difficile* in CAM supplemented with different sugar derivatives, sugar alcohols and L-lactate. Shown are concentrations of TcdA in ng/mg_CDW_ in the supernatant of *C*. *difficile* 630Δ*erm* grown in casamino acids medium (CAM, reference) and CAM supplemented with different sugar derivatives, sugar alcohols and L-lactate. The TcdA concentrations were determined by ELISA after 48 h of cultivation. All TcdA concentrations measured after cultivations in media with different additives were significantly altered compared to that determined in the control medium CAM without any additions (p-value < 0.01).

Next, the extracellular formation of toxin A (TcdA) was determined after 48 h of growth in CAM in the presence of the indicated components (Fig 1). In CAM, 26.4 ng of TcdA per mg cell dry weight (CDW) were secreted. Addition of most of the tested sugars (2 g/L of fructose, glucose, mannose, trehalose, respectively) and the sugar alcohols (2 g/L mannitol or xylitol) resulted in an up to 2.2-fold increased extracellular toxin concentration (p-value < 0.01). L-lactate (500 mg/L) led to a decreased toxin amount in the growth medium (2.2-fold p-value < 0.01); 11.9 ng/mg_CDW_ TcdA compared to growth in CAM. Due to the discrepancies between several previous publications concerning the effects of glucose addition on toxin formation [5, 7, 20, 21], the influence of increased glucose concentration (5 and 10 g/L) was tested. The strongest decrease in secreted TcdA (6.6-fold; p-value < 0.01, 4.0 ng/mg_CDW_) was observed for 10 g/L glucose, followed by 5 g/L glucose (6.3 ng/mg_CDW_), while an 1.4-fold increase in TcdA secretion (p-value < 0.01) was measured (35.7 ng/mg_CDW_) at a concentration of 2 g/L glucose (CDMM). Previously, glucose (0.02–2 g/L) was shown to increase the toxin formation in defined media [7] which is in line with the effect of our CDMM experiment (2 g/L glucose). Additionally, the reduced toxin secretion observed at higher concentrations of glucose (5 and 10 g/L, i.e. 0.5% and 1%) in our defined medium mimicked the toxin reduction previously observed when *C*. *difficile* was grown in the presence of 5 g/L glucose in complex medium which already contained glucose and other mono- and disaccharides [5, 20, 21].

While the effect of the addition of high glucose concentrations on toxin formation was investigated in complex media in detail before [6, 20, 21], the opposite effect caused by low glucose concentration is not understood at the molecular level so far. This requires defined media since complex media with e.g. yeast extract contain an undefined amount of carbohydrates including sugars. In order to understand cellular processes underlying the observed increased toxin production behavior in response to the low glucose concentrations, we compared the transcriptome and the metabolome of cells grown in the presence of 2 g/L glucose with those of cells grown in the absence of glucose. In addition, we conducted metabolome and transcriptome analyses for cultures in the presence of 500 mg/L L-lactate, where a decrease in toxin formation (2.2-fold) was observed.

### Changes in the transcriptome and metabolome induced by 2 g/L glucose supplementation

**Fig 2:**
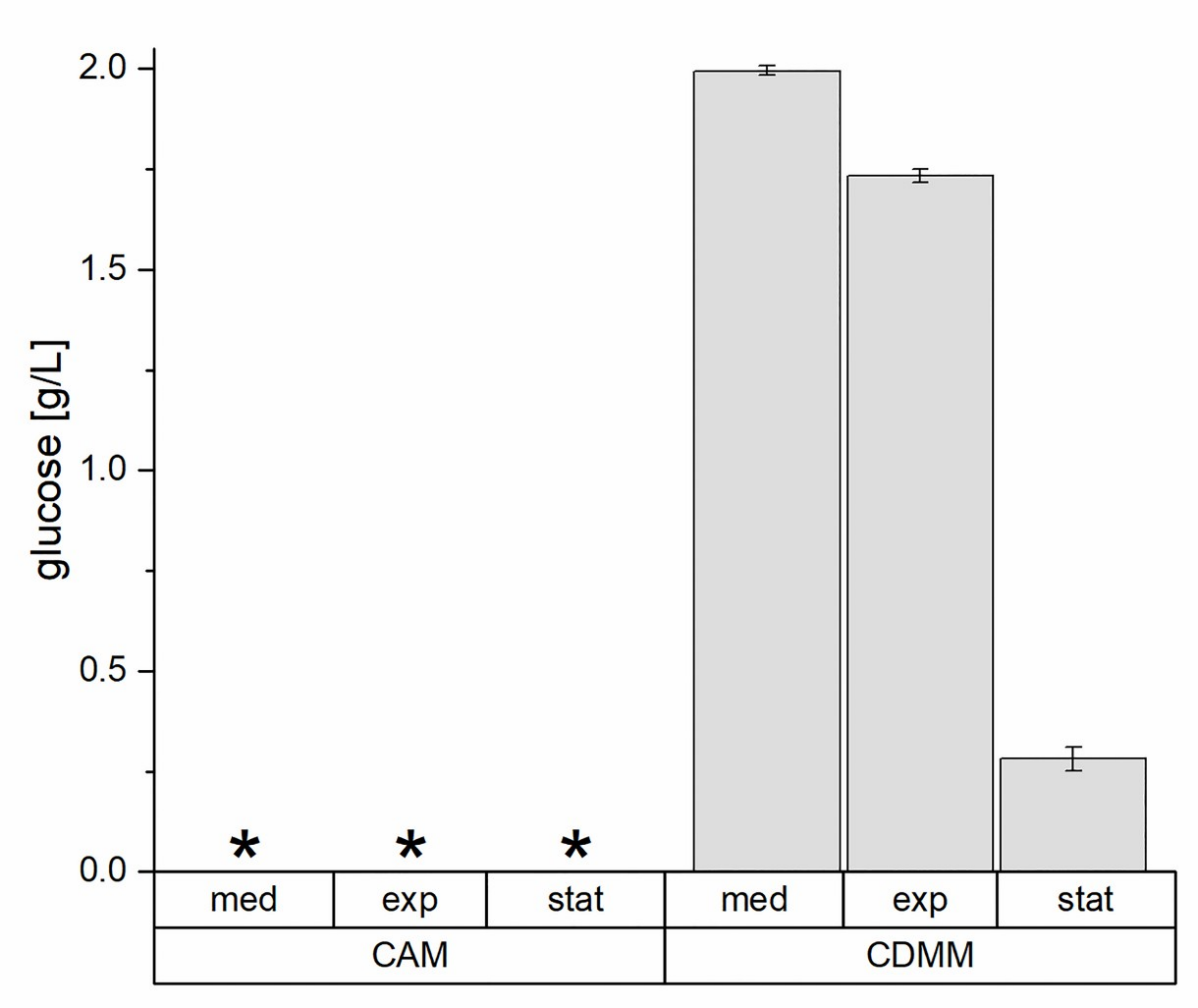
Glucose concentrations in the supernatant of the different cultivations of *C*. *difficile* 630Δ*erm*. Shown are the glucose concentrations of the cultivations in CAM (no glucose addition) and CDMM (addition of 2 g/L glucose) in the growth medium (med), in the samples taken in the middle of the exponential phase (½ OD_max_, exp) and at the beginning of the stationary phase (OD_max_, stat). The concentrations were analyzed by an enzyme assay, results are based on four independent cultivations. *: not detected.

To analyze the effect of glucose supplementation on the metabolism of *C*. *difficile*, we focused on two points in the growth curve. We analyzed cells in the exponential growth phase (½ OD_max_), where previous studies showed that glucose plays only a minor metabolic role compared to amino acids, and in the stationary phase (OD_max_), where the majority of toxin is formed [12, 13]. Supplementation with 2 g/L glucose (CDMM) increased carbon availability about 1.9-fold. After the first half of the exponential growth phase (½ OD_max_) 1.73 ± 0.02 g glucose per L were still present in the supernatant. At the beginning of the stationary phase (OD_max_) most of the glucose was consumed and integrated into biomass (1.65-fold increased when compared to growth in CAM, Fig 2, S1 Fig) and only 0.26 ± 0.03 g/L remained in the supernatant.

**Fig 3:**
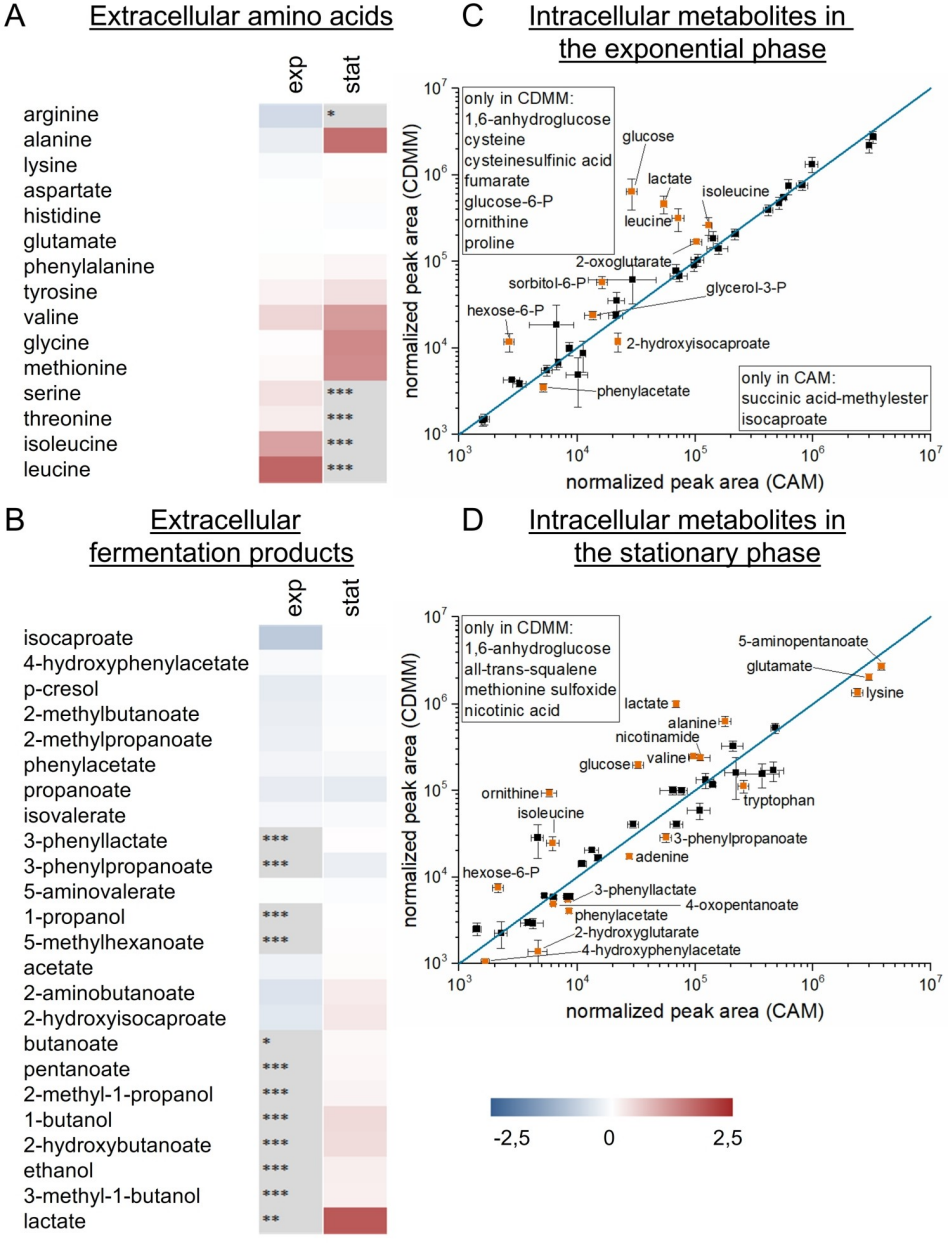
Metabolic data of *C*. *difficile* 630Δ*erm* in CAM and CDMM. Shown are the extracellular metabolites in heatmaps as log2 fold changes of the different amino acids (A) or the fermentation products (B) of the cultivations in CDMM (2 g/L glucose) compared to CAM (no glucose) in the exponential (exp) and the stationary phase (stat). Grey squares indicate not detected metabolites (* not in CDMM, ** not in CAM, *** neither in CDMM nor CAM). The amino acids were analyzed by HPLC-FLD and the fermentation products by GC-MS. The intracellular metabolites were shown as scatterplots from the detected metabolites in the exponential (C) and the stationary phase (D) of cultivations in CAM and CDMM. Known metabolites significantly altered between the two conditions (p-value < 0.05) are labeled in orange. All experiments are based on four independent cultivations.

To compare the metabolism of *C*. *difficile* grown in the presence of 2 g/L glucose (CDMM) and without glucose (CAM), corresponding culture supernatants were analyzed with respect to amino acid consumption as well as the fermentation product profile. At both time points (½ OD_max_ and OD_max_), a reduction of amino acid consumption (especially of branched chain amino acids, glycine, methionine and alanine) due to glucose supplementation was found (Fig 3A). The uptake of preferred amino acids (proline, leucine, isoleucine) differed mainly in the exponential growth phase with fold changes up to 3.3 (p-values < 0.05), while less preferred amino acids showed more pronounced differences in the stationary phase (fold changes up to 3.1, p-values < 0.05). Proline, serine, threonine, isoleucine and leucine were completely used up under both conditions. Only arginine showed a higher consumption in presence of glucose (CDMM, 1.5-fold in exponential phase, p-value < 0.05), and was completely used in the cultivation with glucose until the onset of the stationary phase. The profile of extracellular fermentation products showed rather small differences (Fig 3B, S1 File). In the presence of glucose isocaproate was reduced to 4.8-fold (p-value < 0.05) in the exponential phase, but this effect was completely equalized in the stationary phase. In the stationary phase, we observed a higher abundance of lactate (43.5-fold, p-value < 0.05) and some alcohols like ethanol and 1-butanol (up to 2.4-fold, p-values < 0.05) in the presence of glucose. Overall, the extracellular abundance of fermentation products (isocaproate, isovalerate, 2-methylbutanoate, 2-methylpropanoate) of the Stickland reactions of the branched chain amino acids were reduced in the presence of glucose (from 1.3-fold up to 4.8-fold, p-values < 0.05) in the exponential phase, matching the corresponding amino acid uptake (Fig 3B). Overall, independent of glucose *C*. *difficile* first consumes preferred amino acids (Pro, Cys). After their depletion, it subsequently shifts to use other amino acids in the absence of glucose or to use glucose if available. This is also reflected by the according fermentation products. Thus, the effect of glucose addition is already obvious in the exponential phase but glucose metabolism is more pronounced in later growth phases where toxin production is set on.

Analysis of the intracellular metabolites showed the underlying metabolic changes in the cell. At both time points, concentrations of metabolites of the glycolysis (glucose, glucose-6-P, hexose-6-P) as well as the citric acid cycle (fumarate) were up to 22.4-fold (p-values ≤ 0.05) increased or only found when glucose was present. Again, L-Lactate was significantly increased in the presence of glucose at both time points up to 14.6-fold (p-values ≤ 0.05, Fig 3C). Furthermore, concentrations of some of the amino acids were increased (valine, isoleucine, alanine) up to 4.0-fold (p-values < 0.05) in the stationary phase or some were only found in the presence of glucose (cysteine and proline in the exponential phase), while the fermentation products of the amino acids were found in up to 2.1-fold (p-values < 0.05) higher concentrations without glucose (3-phenylpropanoate, 3-phenyllactate, 4-hydroxyphenylacetate, phenylacetate) (Fig 3D). Overall, the intracellular metabolome revealed a shift from amino acid based metabolism in the absence of glucose to central carbon metabolism in the presence of glucose, and thus, correlated nicely with the observations for the extracellular metabolome.

In addition, the transcriptome in the presence (2 g/L) and absence (reference) of glucose was determined at the same two time points using DNA microarrays. The transcriptome data based on DNA microarrays revealed significant changes (-1 > log2 fold change > 1, p-value < 0.05) for 74 transcripts comparing the exponential phase (expression of 27 genes upregulated) and 510 transcripts comparing the stationary phase samples (expression of 269 genes upregulated).

The highest fold changes (up to 18.9-fold) were found for the expression of genes of the glycolysis (e.g. *bglA5*, *eno*, *gapA*, *gpml*, *pfkA*, *pgk*, *pyk*, *tpi*) and a glycolytic gene regulator (*cggR*). Besides that, the expression of genes of sugar transport, mainly by the PTS-system (e.g. *bglF5*, *ptsG-A*, *ptsG-BC*), the pyruvate formate-lyase (*pfo*) and the cysteine-S-conjugate beta-lyase (*malY*) of the central carbon metabolism were up to 22.3-fold upregulated. The expression of genes from the amino acid degradation, in particular by the Stickland reactions (e.g. *buk*, *grdABCDE*, *iorAGB*, *vorABC*, *ptb1*, *ptb2*), were found to be strongly downregulated in the presence of glucose (up to 11.7-fold, S2 File).

**Fig 4:**
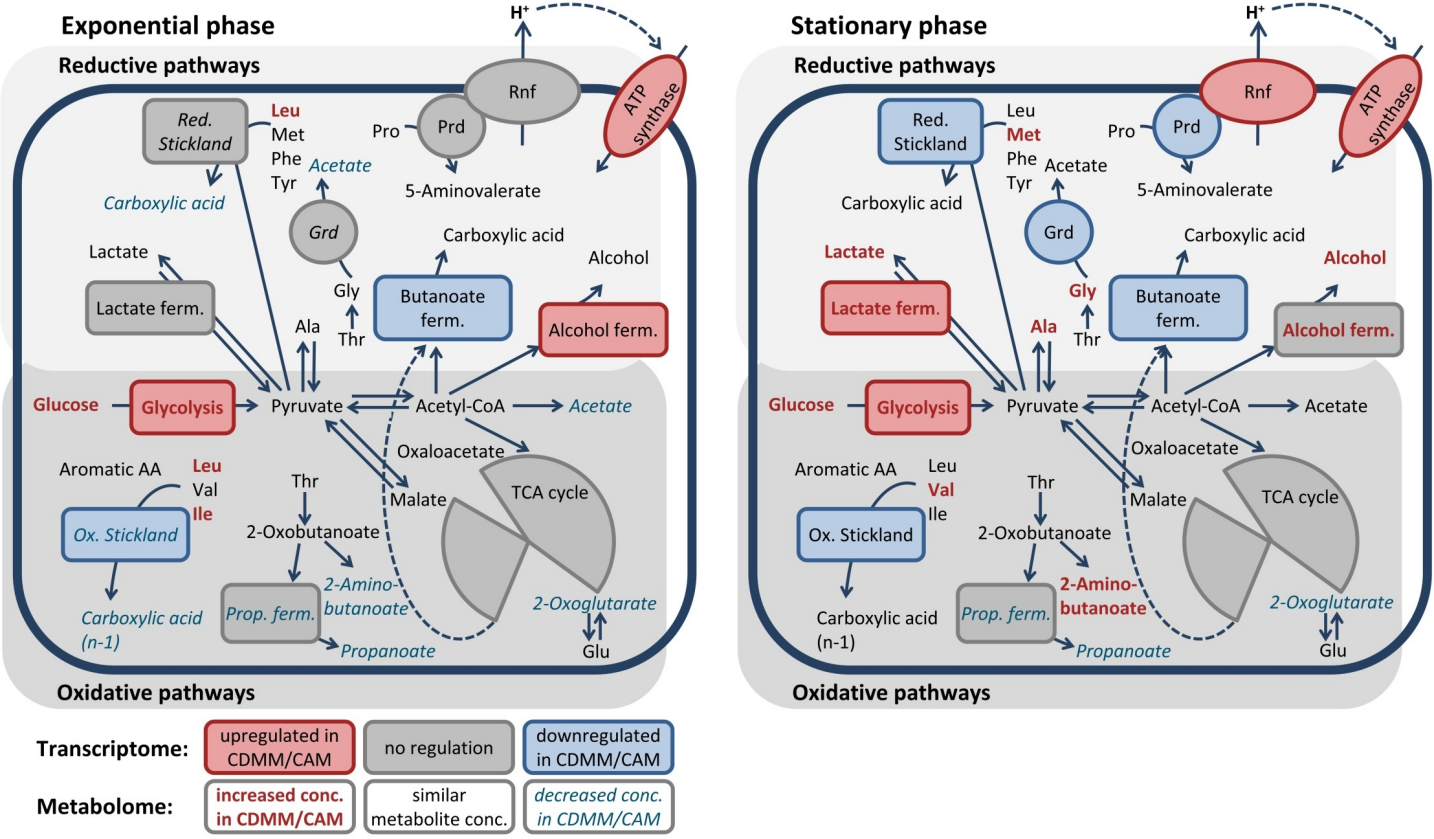
Overview of adaptation of reductive and oxidative pathways in *C*. *difficile* by 2 g/L glucose. Shown are data of transcriptome and metabolome analyses in the exponential (½ OD_max_, left side) and stationary phase (OD_max_, right side) comparing data from CDMM-cultures (2 g/L Glc) with CAM-cultures (without Glc). Pathway boxes represent the transcriptomic data, generally upregulated pathways are shown in red, while downregulated pathways are blue (-1 > log2 fold change > 1, p-value < 0.05). Metabolomic data are represented by the metabolite names and the pathway names inside the boxes, generally increased concentrations are shown in bold and red, while decreased concentrations are italics and blue (fold change > 1.5). Products of Stickland reactions are carboxylic acids with the same length of the corresponding amino acid in the reductive path and one carbon atom shorter than the corresponding amino acid (n-1) in the oxidative path.

Integration of the transcriptome and metabolome data visualized in Fig 4 showed a correlation between the metabolite levels (e.g. glycolysis products, alcohols, carboxylic acids) and the regulation of the corresponding genes (e.g. *pyk*, *pfo*, *adhA*, *buk*, *ldh*). Overall, these observations indicated that the observed toxin production at low glucose concentration might be related to the complex rearrangement of the metabolism with the central carbon metabolism associated fermentation pathways (lactate, acetate, butanoate) as the central element.

When our transcriptome data were compared to those previously obtained for growth at higher glucose concentration some major differences were observed. Previous investigations attributed the decreased formation of toxins in the presence of high glucose concentrations to occur due to a glucose induced regulation of the toxin production [20, 21]. However, complex media were used which already contained undefined amounts of sugars prior to any addition of glucose. Low glucose concentrations pointed towards inactive CcpA and Rex regulatory systems [6, 20–23] which most likely explains the observed contrary behavior concerning toxin production of *C*. *difficile* grown with low and high glucose concentrations.

### L-lactate in the growth medium induces lactate utilization at the transcriptome and metabolome level and changes the NAD^+^/NADH ratio

With respect to food components, L-lactate is mainly found in different dietary products like fermented vegetables, fruits or milk products [24, 25]. In contrast to monosaccharides such as glucose, L-lactate is transported through a large part of the human digestive tract and can be found in the human large intestine [26] where also *C*. *difficile* can be found. Consequently, we aimed to analyze the effect of L-lactate on the metabolism of *C*. *difficile* 630Δ*erm* and its toxin formation in glucose-free medium (CAM). We detected a strong decrease of 2.2-fold for TcdA and 1.8-fold for TcdB (p-value < 0.01) in extracellular toxin formation upon L-lactate addition to the growth medium. However, the presence of 500 mg/L L-lactate (CAM+L) did not affect the growth behavior compared to growth medium without L-lactate addition (S1 Fig).

**Fig 5:**
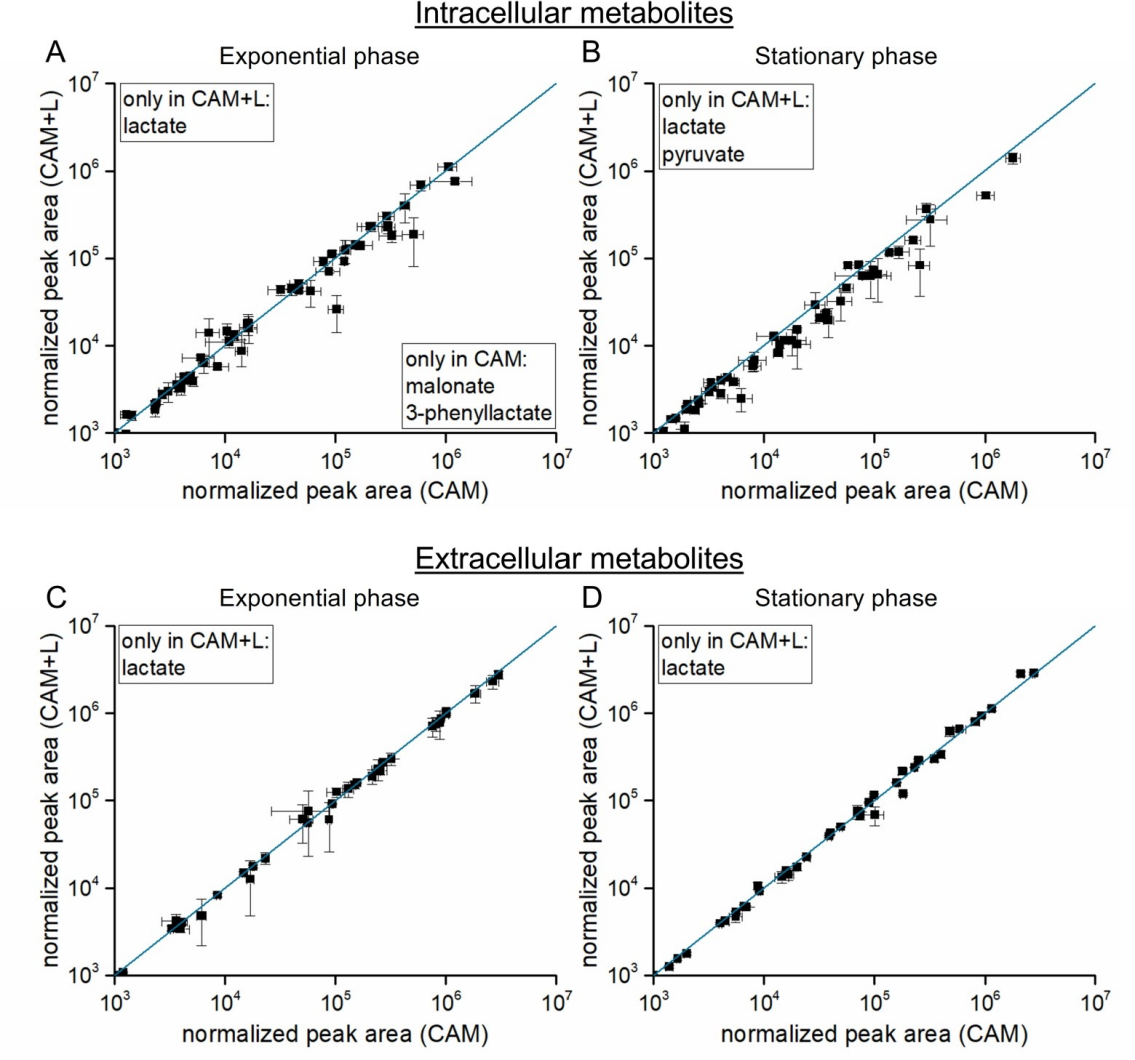
Scatterplots of the intracellular and extracellular metabolome analysis of *C*. *difficile* grown in absence and presence of L-lactate. Shown are the detected intracellular metabolites of the exponential (A) and the stationary phase (B) samples as well as the detected extracellular metabolites of the exponential (C) and the stationary phase (D) samples of *C*. *difficile* 630Δ*erm* cultivated in casamino acids medium without (CAM) or supplemented with 500 mg/L L-lactate (CAM+L). The majority of metabolite concentrations remained unchanged (p-value < 0.05) for the two tested conditions. Metabolites detected only in one condition were shown in boxes. All experiments are based on four independent cultivations.

In order to elucidate the molecular basis for this observation, we analyzed the fate of the L-lactate added to the growth medium at the metabolic level including the major intracellular metabolic changes caused by L-lactate addition. Analysis of the extracellular D- and L-lactate concentration in the stationary phase showed that in summary only about 10% (about 50 mg) of the 500 mg/L L-lactate were consumed while 26 mg/L D-lactate had been secreted into the medium at that time point (Table 1). Interestingly, there were no further significant intra- and extracellular metabolic changes detectable. Exceptions to this general observation were lactate and pyruvate, which were only detected in cultivations with L-lactate, and malonate and 3-phenyllactate, which were only present in the absence of glucose in the exponential phase (Fig 5A–5D, S3 File). Pyruvate, the product of the lactate dehydrogenase reaction, probably resulted from the utilization of the added L-lactate.

**Table 1:** Determination of D- and L-lactate in the culture supernatant of *C*. *difficile*.

	D-Lactate	L-Lactate
	mg/L	mg/L
CAM	nd	nd
CDMM	43 ± 1	nd
CAM+L	26 ± 1	453 ± 14

Quantification of D- and L-lactate in the culture supernatant after 24 h of growth in absence of glucose and L-lactate (CAM), after addition of 2 g/L glucose (CDMM) or 500 mg/L L-lactate (CAM+L); nd, not detectable; based on 4 independent cultivations.

In order to understand the underlying regulatory scenario transcriptome analyses were performed comparing *C*. *difficile* 630Δ*erm* cultivated in CAM+L with cultivation in CAM in the exponential and the stationary phase. Overall, only four genes with significantly increased expression of up to 7.4-fold (p-value < 0.01) were detected (S4 File). These four genes encoding lactate racemase (*larA*), an electron confurcating lactate dehydrogenase (*CDIF630erm_01321*) and the electron transfer flavoprotein (*etfAB*) are all clustered in one operon (*CDIF630erm_01318–01321*, Table 2). The lactate dehydrogenase forms a stable complex with the electron transfer flavoprotein EtfAB that catalyzes the reduction to NADH only in the presence of reduced ferredoxin (Fd^2-^) [27].

**Table 2:** Differently expressed genes in *C*. *difficile* cultivated in absence and presence of L-lactate.

	gene name	Log2 fold change exp	adj. p-value exp	Log2 fold change stat	adj. p-value stat	Annotation (according to [28])
CDIF630erm_01318	*larA*	1.52	5.94E-05	1.42	6.16E-07	lactate racemase
CDIF630erm_01319	*etfB4*	1.21	2.60E-05	1.35	1.90E-06	lactate dehydrogenase (electron bi-/ confurcating), electron transfer flavoprotein beta subunit
CDIF360erm_01320	*etfA4*	2.77	8.14E-07	2.88	9.05E-08	lactate dehydrogenase (electron bi-/ confurcating), electron transfer flavoprotein alpha subunit
CDIF630erm_01321		2.52	5.29E-07	2.59	8.83E-07	lactate dehydrogenase (electron bi-/ confurcating), catalytic subunit

The log2 fold changes of regulated genes (log2 fold change > 1, p-value < 0.01) measured by DNA array in the exponential and at the beginning of the stationary phase from cells cultivated in absence (CAM) and presence of 500 mg/L L-lactate (CAM+L) based on 4 independent cultivations are shown.

**Fig 6:**
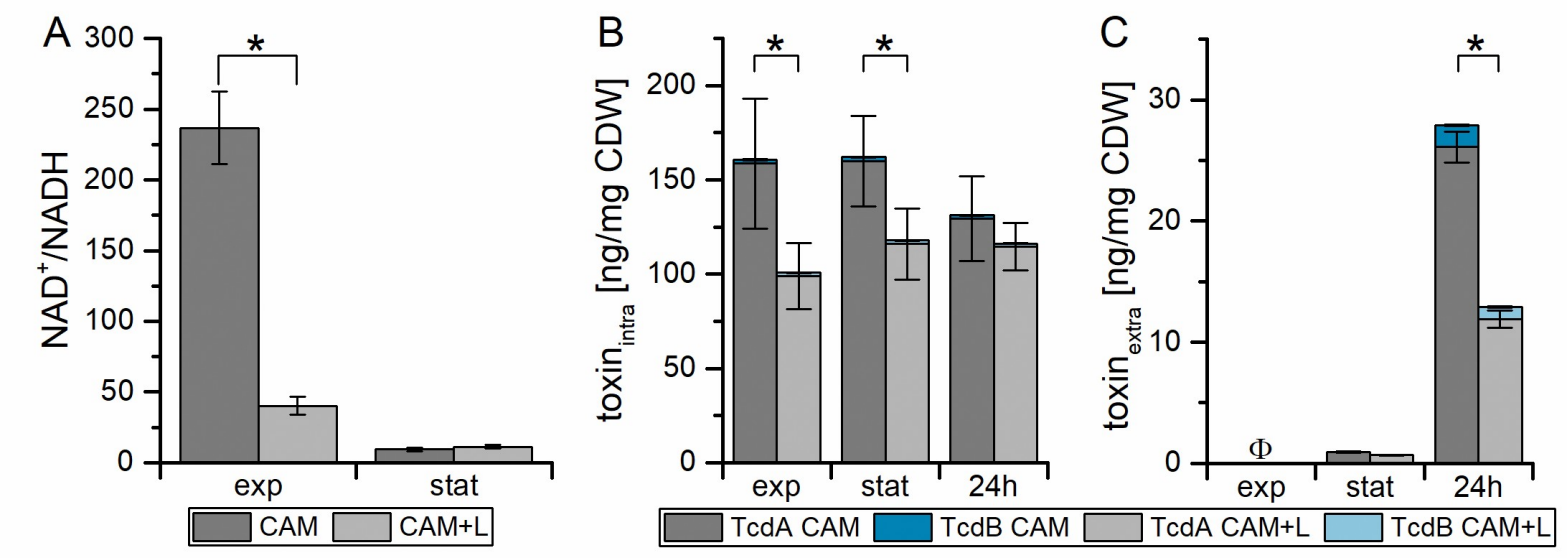
Influence of L-Lactate on NAD^+^/NADH ratio and intra- and extracellular toxin concentration. Shown are the NAD^+^/NADH ratio (A), the intracellular (B) and the secreted (C) toxin A (grey) and B (blue) concentrations in CAM (dark color) and CAM+L (light color). All experiments are based on four independent cultivations. Single values of toxin A and B and of NAD^+^ and NADH are shown in S5 File. *: significant difference in TcdA concentration between the values of CAM and CAM+L (p-value < 0.05), Φ no toxin detected.

Obviously, the addition of L-lactate induced the expression of the lactate utilization operon. Observed metabolic changes were the results of the newly formed proteins. Finally, the shift in metabolism was associated with an inhibition of toxin formation. In order to test the role of the overall energy and redox metabolism in this process, we analyzed the NAD^+^/NADH amounts (Fig 6A) in the exponential and at the beginning of the stationary phase. The NAD^+^/NADH ratio strongly differed between both growth conditions in the exponential phase with a lower ratio in lactate-supplemented medium (CAM+L). In the stationary phase, the values were almost equal. Furthermore, concentrations of intracellular and extracellular toxins A and B were monitored at both time points and after 24 h (Fig 6B and 6C). Intracellularly, toxins were detected in the exponential as well as in the stationary phase. The toxin concentrations decreased significantly up to 1.6-fold in CAM+L (Fig 6B). The lower toxin concentration with lactate was also observed in the extracellular analysis after 24 h (Fig 6C).

## Discussion

### The effect of glucose on metabolism and toxin formation

In recent years, glucose has been found to cause contradictory effects on the toxin production of *C*. *difficile* depending on the overall culture conditions. In some experiments, the addition of glucose led to a reduced toxin production, while other publications showed an induced formation [7, 21, 29].

Already in 1999, glucose was shown to increase the toxin formation in defined media [7] which is in line with the toxin increasing effect comparing 2 g/L glucose to the glucose-free reference in our experiments. Most notably, the branched chain amino acids showed a reduced consumption in the cultivation with glucose in the exponential phase and also of glycine and alanine in the stationary phase. The presence of branched-chain amino acids influences the DNA binding affinities of the global regulator CodY [30]. CodY plays a role in the regulation of about 146 genes, including amino acid biosynthesis, nutrient transport and fermentation pathways. Thus a reduced consumption of branched chain amino acids could have a great influence on the enzyme repertoire and thus on the metabolism [30, 31]. In addition, it was shown that CodY is a repressor of *tcdR* expression, a sigma factor that is responsible for the initiation of the transcription of the toxin genes. In our study, no significant differences in expression of *codY* could be detected.

The reduced or delayed consumption of glycine appears to be linked to the slower consumption of leucine in the earlier growth phase. Besides proline, leucine is the preferred amino acid in the reductive Stickland reaction [12, 13]. Due to the lower consumption of leucine into the exponential phase, the shift to glycine as a reductive substrate occurs later. This is confirmed by the transcriptomic data showing an up to 11.7-fold reduced expression of the genes of the glycine reductase complex in the stationary phase.

The concentrations of the amino acids alanine and arginine revealed an inversely proportional behavior for the cultivation with and without glucose. Arginine was only consumed in the presence of glucose. Alanine was consumed in the absence of glucose while in the presence of glucose it was produced and secreted. Arginine is degraded by a modified Stickland reaction via ornithine and alanine [32, 33]. Later both were found increased in the presence of glucose in our study (S1 File). In addition, arginine is degraded during spermidine biosynthesis, which is a precursor for siderophores [34]. Siderophores are used for iron uptake in bacteria [35, 36], which might indicate an increased iron demand in the cultivation with glucose as seen before [37]. Alanine can be introduced via pyruvate and acetyl-CoA into the fermentation pathway to acetate and butanoate [13, 19]. It might also be used during glucose-free cultivations as a precursor for gluconeogenesis. In the presence of glucose, the increase of alanine concentration might indicate an overflow response due to pyruvate accumulation.

Previous transcriptome investigations for the role of the carbon source glucose and the influence of the transcriptional regulators CcpA and Rex were performed by growing *C*. *difficile* at high glucose concentrations (0.5%, 5 g/L glucose) often using complex media, which already contained an undefined amount of carbohydrates. The obtained results revealed a major overlap of regulatory networks of toxin formation and central metabolism control mediated by CcpA, Rex and PrdE [6, 20, 21]. These results are in good agreement with our observations for *C*. *dfficile* grown in defined medium at higher glucose concentrations (0.5%, 5 g/L, and 1%, 10 g/L, glucose). Furthermore, a reducing effect of tryptose yeast extract (TY) medium containing 1 g/L (0.1%) glucose on toxin formation [6], compared to 5 g/L (0.5%) glucose [6, 20, 21] was also described before. The pleiotropic regulator CcpA promotes the cellular response to rapidly metabolizable carbohydrates. It positively regulates the expression of genes of the PTS uptake systems for various sugars and of enzymes of glycolysis, with the fructose-1,6-bisphosphate aldolase (*fba*) gene as major target. Furthermore, the expression of the *C*. *difficile* toxin genes (*tcdA*, *tcdB*) and of the corresponding genes of their regulators (*tcdC*, *tcdR*) are repressed by CcpA in response to high glucose concentrations in the growth medium. For this purpose CcpA binds to its binding site (Cre) in the promoter of the *tcdA*, *tcdB*, *tcdC* and *tcdR* genes [20, 21]. More recently, the role of the redox-dependent transcriptional regulator Rex was elucidated in *C*. *difficile* which is known to regulate alternative NAD^+^ regeneration pathways in the energy and carbohydrate metabolism and in fermentation pathways depending on the NAD^+^/NADH ratio in gram-positive bacteria [22, 23]. The Rex regulator, responding to the cellular NAD^+^/NADH ratio, is also involved in the control of the central energy metabolism. In the presence of an excess of NAD^+^ the protein is able to bind to promoter elements and to repress the transcription of corresponding genes. Infection experiments revealed that the Rex regulator, most likely indirectly via cooperation with the direct regulator of toxin gene transcription PrdR, is controlling toxin formation in dependence of the redox status of the *C*. *difficile* cells during various modes of Stickland fermentation [23] Looking at the transcriptome and metabolome data obtained in this investigation, it became obvious, that both CcpA and Rex showed no regulation at the glucose concentration used under our experimental conditions (0 and 2 g/l). Thus, the missing control of CcpA or Rex might have caused the induction instead of repression of toxin gene expression explaining the observed phenotypes.

Additionally, there was an increased production of lactate, which could be an overflow reaction due to the high amount of glucose in the medium. Consequently, we aimed to analyze whether the effect of glucose on toxin formation can be (partially) attributed to lactate. As L-lactate is the major form present in humans and is also formed by several probiotic bacteria [38], we decided to use L-lactate instead of D-lactate to evaluate the impact on *C*. *difficile*.

### The addition of L-lactate to the medium induced electron confurcation and altered the NAD^+^/NADH ratio

With the addition of L-lactate to the medium, only the concentration of pyruvate and lactate was altered intracellularly. Both, the intracellular pyruvate or lactate might act as a signal mediating increased/decreased toxin formation. This hypothesis was tested for pyruvate in a complex medium by Dubois et al. [4], where the addition of pyruvate in the stationary phase resulted in a reduced transcription of *tcdA*, *tcdB* and *tcdR*. However, in our transcriptomic analysis we did not detect an influence on the toxin gene expression.

The stereoisomer analysis of the extracellular lactate showed that L-lactate was consumed and D-lactate was secreted. Transcriptomic data revealed an up to 7.4-fold induction of the lactate utilization operon encoding the lactate racemase, dehydrogenase and electron-transferring flavoprotein complex. Firstly, this explains the observed conversion of L-lactate into pyruvate by lactate dehydrogenase. Secondly, lactate-racemase isomerized L-lactate to D-lactate which then got secreted. Thirdly, L-lactate dehydrogenase and the electron-transferring flavoprotein complex (EtfAB) mediate electron confurcation between lactate and pyruvate. Due to the exergonic electron flow from reduced ferredoxin to NAD^+^ and the elevated lactate concentrations, the equilibrium of the endergonic reaction is shifted to pyruvate [27, 28, 39]. This leads to the formation of pyruvate and NADH under physiological conditions in the presence of lactate. Thus, the lower NAD^+^/NADH ratio in the cells cultivated in the presence of L-lactate can be explained by the electron confurcation process, by which NADH is formed. Simultaneously, reduced ferredoxin is consumed which is not available for the generation of the ion gradient by the Rnf complex.

The difference of the NAD^+^/NADH ratio in the exponential phase (Fig 6A) coincides with the lower intracellular toxin concentration in CAM+L cultures. This suggests an influence of the redox status on toxin formation. The redox-dependent transcriptional regulator Rex acts as a repressor of its target genes when the intracellular NADH concentration is low compared to the NAD^+^ concentration resulting in a high NAD^+^/NADH ratio [33, 40], which we observed here under the cultivation conditions in CAM without and with L-lactate (Fig 6A–6C). The transcriptome analyses failed to show significant changes in toxin gene (*tcdA*, *tcdB*, *tcdC*, *tcdE*, *tcdR*) transcription. However, we could show that small changes in the metabolome can have a strong influence on detectable toxin amounts in the culture supernatant. This is probably based on a difference in the stability or secretion of the toxin, as we did not observe major differences in intracellular toxin levels but only in extracellular toxin levels (Fig 6B).

## Conclusion

*C*. *difficile* grown in presence of glucose showed an increased and in the presence of L-lactate a reduced toxin formation. With the addition of glucose, strong differences in the metabolome as well as in the transcriptome were observed. The transcriptome data showed mainly differences in the expression of genes whose products are involved in the transport and degradation of sugar and amino acid degradation in line with the metabolome data. The addition of sugar to the growth medium slowed down the degradation of all amino acids, while fermentation to short-chain acids was increased. With lactate addition, only lactate and pyruvate were changed in the metabolome while only four genes were found to be upregulated under those conditions. These encode a lactate racemase (*larA*), followed by the Etf-genes (e*tfB4* and e*tfA4*) and the gene for a lactate dehydrogenase. The encoded proteins allow the production of pyruvate and NADH from lactate and NAD^+^ by electron confurcation resulting in a drastically reduced ratio of NAD^+^/NADH. These differences suggest that there must be more than one general regulation process for toxin formation in *C*. *difficile*. Glucose supplementation resulted in different toxin formation as a dependency on its initial concentration by different regulators like CcpA, CodY or Rex. The effect of lactate supplementation is apparently more narrow, e.g. by a posttranslational regulation or an effect on the export of the toxin. Our data show the high adaptability of the bacterium and points out the complexity of targeting the toxin formation in the medical field by the application of a certain metabolite or diet. How the determined metabolic and redox changes influence toxin stability and transport remains to be determined.

## Supporting information

S1 Fig Growth curves of *C*. *difficile* 630Δ*erm* cultivated in CAM supplemented with different sugar derivatives. Shown are the mean values of three independent cultivations; lag phase corrected.
(TIF)

S1 File Metabolome data in the exponential and stationary phase in the cultivations in CAM and CDMM. A: Amino acid concentrations determined by HPLC-FLD, B: normalized peak areas of extracellular products based on GC-MS analysis, C: normalized peak areas of intracellular metabolites based on GC-MS analysis.
(XLSX)

S2 File Data of the microarray analysis of *C*. *difficile* 630Δ*erm* in CDMM and CAM in the exponential and the stationary phase. Log2 fold changes in gene transcription measured for *C*. *difficile* 630Δ*erm* in CDMM (2 g/L glucose) in the exponential phase (½ ODmax) and the stationary phase (ODmax) were compared to those in CAM. Shown are the locus tags of the array design [41] as well as the adaptation to Dannheim et al. [28].
(XLSX)

S3 File Data of the gas chromatography mass spectrometry based analysis of the metabolic compounds. Shown are the normalized peak areas of the singe values, the mean values and the standard errors measured for *C*. *difficile* 630Δ*erm* in the exponential (½ ODmax, exp) and stationary phase (ODmax, stat) cultivated in CAM and CAM+L (CAM + 500 mg/L L-lactate). A: extracellular metabolites, B: intracellular metabolites.
(XLSX)

S4 File Data of the microarray analysis of *C*. *difficile* 630Δ*erm* in CAM and CAM+L in the exponential and the stationary phase. Log2 fold changes in gene transcription measured for *C*. *difficile* 630Δ*erm* in CAM+L in the exponential phase (½ ODmax) and the stationary phase (ODmax) were compared to those in CAM. Shown are the locus tags of the array design [41] as well as the adaptation to Dannheim et al. [28].
(XLSX)

S5 File Determined NAD^+^ and NADH concentrations as well as intra- and extracellular concentrations of toxins A and B in the exponential (exp), the stationary phase (stat) and after 24 h of cultivation in cells of *C*. *difficile* 630Δ*erm* cultivated in CAM and CAM+L medium. A: NAD^+^ and NADH concentrations, B: intracellular toxin concentrations, C: extracellular toxin concentrations.
(XLSX)

## Abbreviations

CAM: casamino acids medium
CAM+L: casamino acids medium with 0.5 g/L L-lactate
CDMM: *C*. *difficile* minimal medium (CAM with 2 g/L glucose)
CDW: cell dry weight
GC-MS: gas chromatography/mass spectrometry
Glc: glucose
HPLC: high performance liquid chromatography
log2 fold change: logarithmic fold change with base 2
OD: optical density
